# Current Innovations in the Development of Functional Gummy Candies

**DOI:** 10.3390/foods13010076

**Published:** 2023-12-25

**Authors:** Mohammad Tarahi, Sima Tahmouzi, Mohammad Reza Kianiani, Shiva Ezzati, Sara Hedayati, Mehrdad Niakousari

**Affiliations:** 1 Department of Food Science and Technology, School of Agriculture, Shiraz University, Shiraz 7144165186, Iran; tarahimohammad@yahoo.com (M.T.); niakosar@shirazu.ac.ir (M.N.); 2 Department of Food Science and Technology, School of Public Health, Shahid Sadoughi University of Medical Sciences, Yazd 8916978477, Iran; simaa.tahmouzi@gmail.com; 3 Department of Food Science and Technology, Faculty of Agriculture, Ferdowsi University of Mashhad (FUM), Mashhad 9177948978, Iran; rezakianiani@gmail.com; 4 Department of Food Science and Technology, Faculty of Agriculture, University of Tabriz, Tabriz 5166616471, Iran; sh_ezzati@tabrizu.ac.ir; 5 Nutrition Research Center, School of Nutrition and Food Sciences, Shiraz University of Medical Sciences, Shiraz 7193635899, Iran

**Keywords:** functional foods, novel formulations, confectionary industry, soft candy, jelly candy

## Abstract

Nowadays, consumers are aware of the necessity of following a healthy diet and there is demand for natural and nutritious food products, especially for children. Consequently, new trends in the food industry are focused on the development of foods with low levels of sucrose and artificial additives (e.g., flavors and colorants), as well as high antioxidant, protein, and fiber content. On the other hand, some consumers demand vegan, halal, and kosher-certified food products. In this regard, conventional confectionary products such as gummy candies (GCs) are increasingly losing their popularity. Therefore, the development of plant-based and functional GCs has gained the attention of researchers and manufacturers. This review highlights recent innovations in the development of GCs with alternative gelling agents and sweeteners, natural flavors and colorants, and the incorporation of medicines, fiber, protein and antioxidants into GCs. Additionally, it summarizes their effects on the techno-functional, sensory, and nutritional properties of GCs.

## 1. Introduction

In recent decades, consumer demand in the field of food production has changed significantly, as they have become increasingly aware of food quality and the health benefits associated with different food products. Nowadays, foods are intended not only to satisfy hunger and provide necessary nutrients for humans, but also to prevent diseases related to nutrition and improve the physical and mental health of consumers. In this regard, it is necessary to develop novel functional foods to meet these demands [1]. The term “functional food” was first used in Japan in the 1980s for food products enriched with certain compounds that have beneficial physiological effects, such as pre- and probiotics, as well as cholesterol-lowering substances. These types of food products can be consumed in order to improve the general condition of the body, reduce the risk of some diseases, and can even be used to treat some illnesses [2]. In 2014, the Functional Food Center (FFC) defined functional food products as “natural or processed foods containing effective and non-toxic amounts of bioactive compounds, which provide a clinically proven and documented health benefit through the use of specific biomarkers to prevent, manage, or treat chronic diseases or their symptoms” [3]. However, despite these different definitions, scientists generally agree that functional foods contain bioactive substances that provide health benefits for the human body beyond the usual dietary supplements.

The confectionery industry has a wide range of products with approximately USD 298.23 billion global market value in 2021, which is projected to expand at a compound annual growth rate (CAGR) of 4.3% from 2022 to 2028 [4]. Among these products, soft confectionery products have attracted the attention of consumers in all age groups, especially children, due to their unique texture, appearance, and taste [5]. Gummy candy (GC) is one the most popular types of soft confectionery products, which is also known as pastille, gummy, jelly candy, gummy jelly, and so on, with a global market value of USD 21.40 billion in 2022 [6,7]. GCs are traditionally made from high amounts of sucrose and glucose syrup combined with gelling agents, commonly known as gelatin, along with artificial additives such as flavors and colors. The raw materials are first heated and then poured into the molds with different shapes and sizes. After molding, GCs are cooled and dried by air convection in chambers or tunnels to achieve a dry-sticky texture in the shortest possible time, with a relatively high-water content varying between 15 and 30% [8]. Although gelatin offers a variety of functional properties in GC preparation, such as firmness, consistency, and gelling properties, some consumers demand vegan, halal, and kosher-certified food products [9]. Besides, these popular foodstuffs are often of low nutritional value and high sugar content, which may increase the risk of several chronic diseases, such as obesity, tooth decay, hyperglycemia, and type 2 diabetes. In addition, the overconsumption of low-nutrition confections by children is gradually increasing, which has become a serious concern for their parents [10]. In this respect, the development of new formulations can be a promising step for GC lovers in order to provide desirable health benefits and pleasant sensory attributes to certain types of diets.

In recent years, the incorporation of alternative gelling agents, sugar substitutes, natural flavorings and colorants, antioxidants, and other value-added ingredients into GC formulation has revolutionized the confectionery industry, offering exciting new possibilities for taste, texture, and nutritional values to meet consumer demands. Therefore, this study aims to comprehensively investigate the preparation and development of GCs based on these innovative formulations.

## 2. Preparation of GCs Based on Novel Formulations

**Figure 1:**
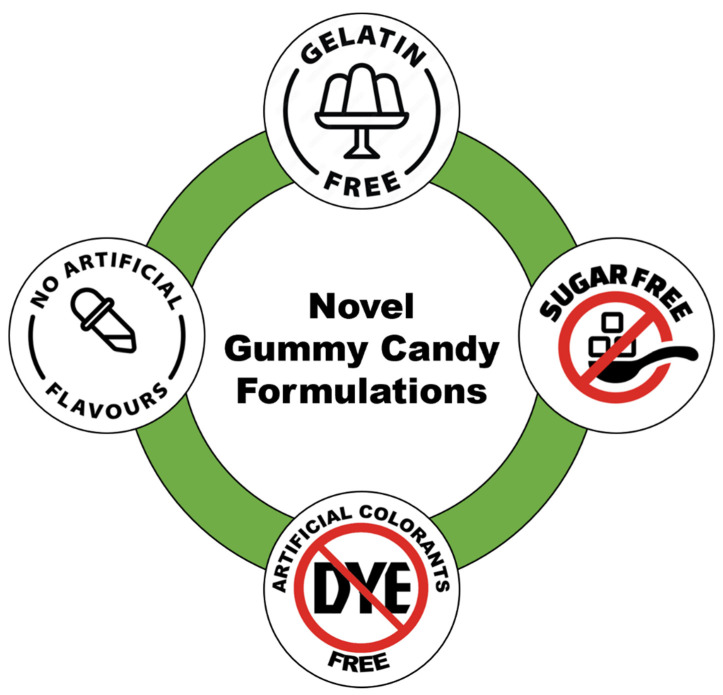
A schematic of GC preparation based on novel formulations.

Novel formulations have opened doors for customization and personalization of food products, by introducing a variety of value-added ingredients, flavors, and other combinations. One significant aspect in the preparation of novel GCs is the incorporation of gelatin alternatives, such as pectin and agar gum, which allows manufacturers to create vegan gummies with unique textures and sensory properties. Furthermore, sugar-free formulations have become more prevalent, allowing individuals with dietary restrictions to indulge in these delightful treats without compromise [11]. Manufacturers have also begun to explore alternatives to artificial colors and flavors, opting for natural fruit extracts and plant-based dyes. This shift toward more natural ingredients not only enhances the appeal of GCs but also aligns with the growing demand for healthier confectionary foods (Figure 1). 

### 2.1. Alternative Gelling Agents

As the demand for vegan and vegetarian-friendly options continues to grow, alternative gelling agents such as pectin, gums, and starches have gained more attention [12]. However, the incorporation of these components in the preparation of GCs requires careful consideration of several factors, including optimization of formulation, selection of appropriate gelling agent, and pH adjustment, as well as their effects on texture, flavor, and shelf life of the final product. Therefore, it is important to evaluate various functional and physicochemical aspects of the alternative gelling agents to adjust the desired formulation [13]. For example, pectin requires an acidic environment to activate its gelling properties, so it may need to be used with acidic ingredients, such as lemon juice. On the other hand, agar gum has high gelling strength and can create a firmer texture in GCs compared to gelatin, so it may be used less than other gelling agents [14]. 

Pectin is one of the most popular alternative gelling agents in the production of jams and jellies, which is commonly derived from different fruits. In order to use pectin as a gelling agent in GCs, it is first dissolved in water and then heated before being added to the candy mixture. Pectin has a different gelling profile compared to gelatin, and it can create a softer and more delicate texture for GCs. However, pectin may require more sugar than gelatin to achieve the desired sweetness and flavor. Besides, DeMars and Ziegler [14] showed that the gelatin-pectin combination at different levels (i.e., 3.0, 4.5, and 6.0% gelatin and 0.5, and 1.5% pectin) can significantly affect the texture of GCs. Here, the addition of pectin reduced the strain at the fracture of gelatin gels. In addition, Renaldi et al. [15] showed almost high sensory score ranges (6.9–7.5) for GCs formulated with 1.42:8.58% pectin-gelatin mixture. Guar gum is another natural polysaccharide derived from the seeds of the guar plant, which has been widely used in the food industry for its unique thickening, stabilizing, and emulsifying properties [16,17]. Guar gum can swell and disperse in both cold and hot water; while, other gums such as locust bean gum, disperse only in hot water [18]. Additionally, guar gum can improve the textural and color parameters of gummy candies. Thus, it can be considered as a promising gelling agent in candy production. In this regard, Dinesh Kumar et al. [19] found that the addition of guar gum in GC production resulted in novel products with better texture and brighter color, which were preferred by the panelists. Therefore, guar gum can be employed as an alternative gelling agent, to achieve the desired appearance, texture, and mouthfeel. 

Agar gum and carrageenan are other important alternative gelling agents, which can be obtained from seaweeds. Similar to pectin, to use agar gum in GC production, it must first be dissolved in water and heated to activate its gelling properties [20]. However, too much agar gum can create a rubbery texture in GCs; hence, careful testing and experimentation are necessary to achieve the desired results. Ge et al. [21] found that incorporating different hydrocolloids, such as pectin and agar gum, into gelatin-based GCs had a synergistic effect on the gel network. These effects are mainly impacted by their nature and ratio to gelatin in the GC formulation. On the other hand, carrageenan has a gelling profile similar to gelatin and can create a delicate texture in GCs. However, it is more challenging to work with carrageenan compared to other alternative gelling agents and may require additional ingredients, such as calcium, to activate its gelling properties. More recently, Song et al. [22] evaluated the effects of carboxy methyl cellulose (CMC) on the texture properties and storage stability of kappa-carrageenan (KC) GCs. The researchers found that the addition of CMC to KC formed flexible GCs with low fragility and limited water syneresis during storage. This study also found that KC promoted side-by-side intermolecular aggregation of CMC helices through hydrogen bonds, which stabilized a denser network structure. These results provide preliminary evidence for utilizing hydrocolloids to adjust texture and control water migration in KC gels to promote vegan GCs. In another study, Hamka [23] investigated the physical and sensory properties of dragon fruit GCs with the addition of carrageenan at different concentrations. The results showed that the water and ash content of GCs fulfilled the required standards at all carrageenan concentrations. Also, the organoleptic test represented that the panelists preferred GCs formulated with 3% carrageenan. 

Alginate is an important polysaccharide derived from seaweeds, which gained much attention in the food industry due to its unique properties, such as gel-forming ability, non-toxicity, and low cost. In GC production, alginate can be used as both a gelling agent and an encapsulation material. For instance, Ali et al. [24] showed the successful production of strawberry and red beetroot GCs incorporated with alginate with favorable nutritional and textural properties. Also, the possible application of cold-set gelation technique was evaluated for GC production by de Avelar and Efraim [25] using a mixture of alginate and pectin. In terms of textural properties, alginate/pectin-based GCs showed a higher hardness value (16.2 N) compared to pectin-based GCs (6.2 N). However, no significant difference (*p* > 0.05) was observed between alginate/pectin and pectin GCs for all sensory attributes, proving the cold-set gelation method as a sustainable alternative technique for the production of GCs.

Starches are also widely used in various sectors of the food industry as a thickening agent, texturizer, and binder to provide the desired texture and mouthfeel for final food products, such as GCs [26,27]. For instance, corn starch has a high water-holding capacity, which can help to improve the moisture content and overall texture of candies. Marfil et al. [28] investigated the microstructure and texture of GCs incorporated with gelatin and corn starch in different ratios (i.e., 10:0; 9:1; 8:2; 7:3; 0:10). The results showed that as the concentration of starch increased, GCs exhibited higher adhesiveness and stringiness values, which is favorable for GC production. In another study, Pereira et al. [29] evaluated the physical and sensory characteristics of GCs containing 12 and 16% acid-thinned cassava starch compared to control GCs with 8% acid-thinned corn starch. The GCs formulated with 12% cassava starch and 8% corn starch presented similar hardness, chewiness, and sensory attributes, while the incorporation of 16% cassava starch almost doubled textural values and decreased sensory scores. These results showed the possible production of GCs with acid-thinned cassava starch.

Overall, various alternative gelling agents can be successfully utilized in GC production, which promises the production of novel vegan GCs. However, the application of these gelling agents may also have some drawbacks. For instance, some alternative gelling agents require more sugar compared to gelatin to achieve the desired flavor, which may be dangerous for diabetic patients and should be carefully optimized in GC formulation.

### 2.2. Alternative Sweeteners

GCs have been a beloved treat for generations, with traditional recipes using sugar as the primary sweetener. However, with the rise of health concerns related to high sugar consumption, alternative sweeteners (e.g., stevia, erythritol, xylitol, honey, etc.) have become increasingly popular in the food industry. Incorporating alternative sweeteners in the preparation of GCs requires careful consideration of several factors, including the choice of sweetener, recipe modification, and their effects on the texture, flavor, and shelf life of the final product [30]. 

One of the most popular alternative sweeteners is stevia. It is a plant-based sweetener that is 200 to 400 times sweeter than sucrose but has no calories. When using stevia as a sweetener in GCs, it is important to remember that it has a different flavor profile than sugar and may require additional adjustment of the recipe to achieve the desired flavor. Additionally, because stevia is much sweeter than sugar, less may be required to achieve the same level of sweetness. The primary components responsible for stevia’s sweetness are steviol glycosides, particularly rebaudioside A and stevioside [31]. Numerous studies have explored the application of stevia as a sweetener in GCs, demonstrating its feasibility and benefits. For instance, Rivero et al. [32] conducted research to develop stevia-sweetened GCs with an emphasis on the sensory characteristics. The study concluded that the low-calorie GCs formulated with stevia exhibited desirable sensory attributes (e.g., texture, flavor, and sweetness) with high consumer acceptability, indicating the potential of stevia for replacing sugar in GC production. In another study, Aranda-Gonzalez et al. [33] investigated the effects of sugar substitution with 20–100% stevia on the physicochemical and sensory properties of GCs. They found that the GCs with 60% stevia exhibited similar physicochemical properties to those made with 100% sugar. Moreover, the sensory evaluation indicated that stevia-sweetened GCs were well-liked by consumers. In addition, Samakradhamrongthai and Jannu [34] explored the formulation of novel GCs using a combination of stevia and other alternative sweeteners (i.e., xylitol, corn syrup, and erythritol). The study aimed to enhance the sweetness profile while minimizing any potential off-flavors associated with stevia. The results indicated that blending stevia with other sweeteners, such as erythritol or xylitol, improved the taste and overall quality of the gummy candies, offering a promising solution for the GC industry.

Another popular alternative sweetener is erythritol. Erythritol is a sugar alcohol that is derived from corn. It is approximately 70% as sweet as sugar and has a similar texture to sugar. Also, Gan et al. [11] showed that the incorporation of erythritol into GCs can effectively reduce the glycemic index of the final products from 81.9 to 49.9%, indicating its potential to be used in the manufacturing of food supplements for diabetics. Besides, Le et al. [35] evaluated the possible application of erythritol in GC production using three-dimensional (3D) printing technology. The results indicated that when erythritol is regularly added from 0 to 30 g into the printing materials, surface smoothness is dramatically improved. However, the gel structure started to show partial bottom adiposity with the addition of 40 g erythritol, which may be due to the inability of hydrogen bonding between polyols to form a stable gel, thereby weakening gel strength. This research can serve as a basis for the development of 3D printed in the future. Furthermore, it should be noted that erythritol may also have a cooling effect in the mouth when consumed in large quantities, which can affect the overall taste of GCs [36]. Therefore, its proper concentration should be optimized in GC formulation. Xylitol is another commonly used sugar alcohol in GC production. Xylitol has a similar sweetness and texture to sugar and can be used in the same proportion as sugar in most recipes [37]. In this regard, Čižauskaitė et al. [38] conducted a comprehensive analysis of the bioactive compounds in xylitol-sweetened GCs. The study revealed that xylitol not only acted as a sweetener, but also contributed to improve the overall nutritional profile of the GCs. Xylitol supplementation increased the content of polyphenols and other beneficial compounds, potentially offering additional health benefits beyond sweetness. As further research and advancements continue, incorporating xylitol in GC formulations can lead to healthier confectionery options that satisfy consumer cravings while addressing oral health concerns and dietary requirements. However, xylitol can be toxic to pets, so it is important to label the candies properly and keep them out of reach of animals [39].

Honey is another alternative sweetener that can be used in GC recipes. Honey is a natural sweetener with a unique flavor profile that can enhance the sensory attributes of candies. Utilizing honey as an alternative sweetener in GCs presents an opportunity to create confectionery products with natural, nutrient-rich, and potentially beneficial options [40]. However, honey is much sweeter than sugar and can also affect the texture of candies. In this regard, Rivero et al. [41] examined the effect of honey on the glycemic response to GCs. This study compared the glycemic index of honey-sweetened GCs to those sweetened with sucrose. The results indicated that GCs sweetened with honey had a lower glycemic index, which suggests a potentially more favorable effect on blood sugar levels, making them a suitable option for individuals monitoring their glucose levels. Additionally, pure honey is difficult to work with and may require additional adjustments to the recipe to achieve the desired results. For instance, Gunes et al. [5] highlighted that honey’s high moisture content could impact the texture and stability of GCs, potentially leading to stickiness or softening. Additional adjustments, such as modifying the gelatin or pectin content, may be necessary to overcome these challenges and achieve the desired GC consistency. However, some confectionery manufacturers have reported that honey may adversely affect the texture and shelf life of GCs and make the final products stickier [42].

Other sweeteners such as isomaltulose, maltitol, and isomaltitol, can also be used in the GC industry. For instance, Gok et al. [43] substituted glucose syrup with maltitol syrup in the formulation of GCs and investigated the effect of different bulking agents (sucrose, mannitol, and soluble wheat fiber) on the physicochemical and sensory attributes of GCs. They found that soluble wheat fiber was the best bulking agent for the GCs with maltitol; while mannitol caused negative changes in the quality of GCs. Besides, Periche et al. [10] demonstrated that the combination of 30% isomaltulose and 70% fructose can be a suitable way to replace traditional sugars in functional GCs with the desired features in terms of optical (e.g., a*, b*, and L* values) and mechanical properties (e.g., springiness, hardness, gumminess, and cohesiveness). Moreover, Jiamjariyatam [44] showed that the toughness and hardness of GCs were significantly decreased by the incorporation of 20, 30, 40, 50, and 100% isomaltulose. Similarly, Ünal and Arslan [45] reported lower hardness, flexibility, and moisture content for isomaltitol-containing GCs than conventional sugar-containing samples. However, the substitution of sugars with isomaltitol negatively affected the sensory acceptability of GCs due to their opaque appearance.

Another approach involved using natural sugar sources like grape, mulberry, and carob molasses to replace sugar syrup and artificial additives in GC production. In this respect, Kurt et al. [46] found that the type of incorporated molasses can significantly affect the texture and appearance of GCs. Grape molasses-sweetened candies had higher thermal stability and lower temperature sensitivity due to their higher total sugar content compared to GCs incorporated with mulberry and carob molasses. On the other hand, sugar can act as a natural preservative, while alternative sweeteners may not have the same effect [36]. Therefore, it may be necessary to add additional preservatives or adjust the packaging of the final products in order to provide sugar-free GCs with a suitable shelf life.

As a result, it is important to adjust the GC recipe according to sweetness and other physicochemical and structural properties of the alternative sweeteners (e.g., melting temperature, particle density, and particle size). The use of alternative sweeteners can also affect the texture and shelf life of GCs. In addition, some alternative sweeteners may have a different mouthfeel or aftertaste than sugar, which could affect the overall flavor of GCs. On the other hand, some studies showed that the consumption of artificial sweeteners (e.g., saccharin, sucralose, aspartame) in food formulations may cause glucose intolerance by inducing compositional and functional changes in the gastrointestinal microbiota [47]. Therefore, it may be necessary to analyze different alternative sweeteners more comprehensively to find one that works best for the GC production. 

### 2.3. Natural Flavors and Colorants

Natural flavors and colorants have become increasingly popular in the food industry as consumers become more conscious of what they are eating. Therefore, incorporating natural ingredients in the preparation of GCs based on novel formulations can enhance the overall flavor and visual appeal of candies [48]. However, careful attention must be paid to the selection and application of natural food flavors and colorants to ensure their compatibility with the conventional recipe and to provide the desired texture and shelf life for GCs.

Fruits can create a wide variety of flavors in GCs, from strawberry and raspberry to orange and lemon. When using fruit flavors, it is important to select natural flavorings derived from the actual fruit rather than artificial or synthetic ones. Whether it is the tangy burst of citrus, the tropical sweetness of mango, or the juicy essence of berries, fruit flavors can add excitement and variety to GC offerings. Generally, consumers appreciate flavors derived from real fruits, and the overall sensory appeal of GCs is enhanced even with the addition of small amounts of fruit flavors. In this regard, Bagautdinov et al. [49] investigated the sensory attributes and consumer preference for GCs flavored with different fruits, including strawberry, orange, and pineapple. The study found that the GCs with fruit flavors were highly favored by consumers, especially strawberry- and orange-containing GCs. Overall, the natural fruit flavors contributed to the overall enjoyment and acceptance of GCs. Moreover, Ali et al. [24] investigated the effects of strawberry as a natural flavoring agent and red beetroot as a natural colorant on the textural and sensory properties of GCs. The study revealed that GCs with natural colors and flavors exhibited variations in texture, such chewiness and firmness. For instance, GC containing a large amount of beetroot was harder than the control sample, which may be related to the increase in fiber and total soluble solids (TSS) of the sample. Also, the incorporation of specific fruits allowed for tailoring the mouthfeel of GCs, enhancing the overall sensory experience for consumers. 

Incorporating natural colorants in the preparation of GCs can also enhance their visual appeal. Natural colorants can be derived from various sources, including fruits and vegetables [50]. For example, the extract of the *Clitoria ternatea* L. flower has been used as a natural colorant for the preparation of novel GCs. The study found that the incorporation of 30 g of *C. ternatea* extract improved the color and appearance of GCs, making them more appealing to consumers [51]. In another study, Casas-Forero et al. [52] showed that GCs become darker and more reddish with the addition of freeze-dried blueberry juice due to the presence of anthocyanins. Also, the enriched samples exhibited significantly higher total bioactive compounds (773.3 mg GAE/100 g) and antioxidant activity (4585.4 µM TE/100 g), compared to conventional GCs (233.4 mg GAE/100 g and 782.9 µM TE/100 g, respectively). These results indicate that fruit flavors not only improve the taste, aroma, and appearance of GCs, but also offer potential health benefits through their antioxidant properties. In another study, de Oliveira Nishiyama-Hortense et al. [53] investigated the applicability of grape juice as a natural colorant in GCs and determined their anthocyanin content and sensory properties to evaluate the quality of the GCs. The GCs presented a shiny appearance with a uniform purple color, retention of anthocyanin was 41% and sensory scores were satisfactory.

Nevertheless, many natural colorants, such as betanin, are sensitive to heat and pH, and their color may deteriorate over time. Additionally, their sensitivity to oxidation and low bioaccessibility limits their applications in food products. Encapsulation is a promising approach to overcome the shortcomings of natural colorants in GCs. In this regard, Amjadi et al. [54] loaded betanin in liposomal nanocarriers to improve its stability in GCs. The antioxidant activity and stability of betanin nanoliposomes were considerably higher than those of free betanin in GCs and their sensory parameters did not show significant differences. Consequently, betanin nanoliposomes can be considered a suitable natural colorant for gummy candies. 

However, the selection of appropriate natural food flavors and colorants is very critical in order to provide favorable techno-functional properties without negatively affecting the texture of the final products. For instance, the incorporation of some natural ingredients, such as fruit purees, increases the moisture content of the system, which can finally affect the texture of GCs [55]. Therefore, it may be necessary to adjust the recipe to account for these changes by using less liquid or adding additional thickening agents to the formulation of GCs. Another consideration for using natural food flavors and colorants is their potential impact on the shelf life of GCs. Natural ingredients may not have the same preservative properties as artificial ingredients and may reduce the shelf life of GCs [52]. To ensure the best results when incorporating natural food flavors and colorants in the preparation of GCs, it may be necessary to adjust the recipe multiple times to achieve the desired flavor, color, and texture. 

In conclusion, incorporating natural food flavors and colorants in the preparation of GCs based on novel formulations can enhance the overall flavor and visual appeal of GCs, as well as their health-promoting properties. However, careful consideration must be given to the selection and application of these natural ingredients to ensure their compatibility with conventional recipes and to maintain the desired texture and shelf life of the GCs.

## 3. Development of GCs as a Functional Food

**Figure 2:**
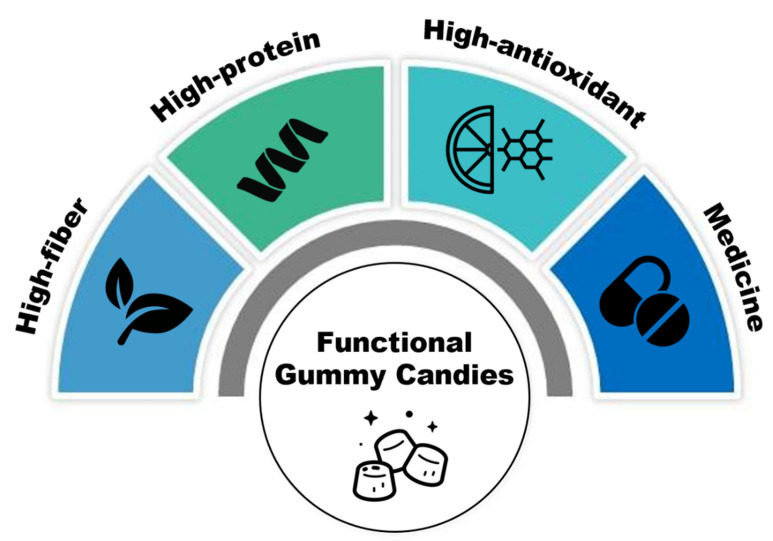
A schematic of GC development as a functional food.

Conventional GCs contain high amounts of absorbable carbohydrates and low levels of health-promoting compounds, which have raised many health concerns for consumers. As a result, enrichment with different fibers, proteins, antioxidants, minerals, vitamins, and probiotics is a suitable solution to improve the nutritional value of these products (Figure 2).

### 3.1. High-Fiber GCs

Dietary fibers (DFs) include non-digestible forms of carbohydrates, originating from plant-based foods. They can also be categorized into soluble and insoluble fibers according to their solubility in water. The high nutritional value of DF has been well documented over the years and it is widely accepted that DF intake is associated with several health-promoting effects, including protection against cardiovascular and cancer diseases, reduction of blood glucose and cholesterol, regulation of intestinal function, and promotion of gut health [56]. Current DF intake recommendations for adults in most European countries and in the United States are between 30 and 35 g/day for men and between 25 and 32 g/day for women. However, according to the European Nutrition and Health Report, DF intake was below the recommended levels in most European countries [57]. Therefore, the incorporation of DFs into food products is essential to meet such recommendations.

Cappa et al. [58] investigated the effects of adding grape skins with different particle sizes (i.e., small, medium, large) on the physicochemical properties of fruit GCs. The enriched GCs contained more than 60 g fiber per kg product weight, which can be claimed as “high in fiber”. Also, fiber-fortified GCs showed better antioxidant and textural features that remained stable within processing. Similarly, Ali et al. [24] showed that the DF content of GCs increased from 0.04 g/100 g to 1.02, 0.92, and 0.90 g/100 g with the addition of 25–75% strawberry and red beetroot. In a further study, Hariadi [59] evaluated the effects of carambola starfruit (*Averrhoa bilimbi*) and papaya (*Carica papaya*) fruits on the physicochemical properties of GCs. The results revealed that carambola starfruit contained a higher amount of DF (2.8 g/100 g of fresh fruit) compared to papaya (1.8 g/100 g of fresh fruit). Therefore, by increasing the proportion of carambola starfruit in the GC formulation from 25 to 75%, the DF content of GCs increased from 0.94 to 0.97 g/100 g. These findings introduce novel fruit-based GCs with favorable fiber content.

Non-starch polysaccharides, such as inulin and konjac glucomannan (KGM), are other important DFs that can be utilized in various food products as non-digestible and health-promoting ingredients. Inulin is composed of fructose units linked by β-(2-1)-_D_-fructosyl-fructose bonds, improving techno-functional and nutritional properties of foodstuffs [60]. In this regard, Delgado and Bañón [61] investigated the effect of substitution of starch with inulin on the physicochemical, texture, and sensory properties of GCs. Inulin-containing GCs provided a slightly softer, springier, and stickier texture. In addition, the incorporation of inulin can not only improve the technological and sensorial properties of GCs, but also increase the DF of samples with potential prebiotic activity. Similar results were also presented by Cedeño-Pinos et al. [62] with the addition of β-fructan fibers (e.g., chicory inulin and fructooligosaccharides) into the GC formulations as alternative ingredients to starches and sugars. In another study, Fernandes et al. [63] reported that the waist circumference and hunger/appetite of forty-two overweight individuals decreased after the consumption of KGM-enriched GCs for fourteen days. These results indicate that the incorporation of KGM into the GC manufacturing process can significantly prevent the development of cardiovascular diseases in overweight and obese individuals. 

### 3.2. High-Protein GCs

Proteins are important ingredients in the human diet due to their essential role in immune responses, repairing damaged cells, and maintaining muscle mass [64]. However, according to previous studies, more than 10% of people suffer from malnutrition and protein-energy undernutrition (PEU), especially in the developing countries of Asia and Africa. It is estimated that 149.6 million children under the age of five are malnourished, which causes about 50% of child mortality globally [65,66]. Therefore, the development of high-protein food formulations is necessary to increase the protein intake of consumers worldwide. 

In this respect, some researchers evaluated the possible application of legume proteins into GCs. Siegwein et al. [67] evaluated the textural, rheological, and sensory properties of starch-based confections enriched with 33, 50, and 66% soy protein isolate (SPI). According to the results, SPI could reduce the hardness, cohesiveness, and gumminess values of GCs by the disruption of starch gel network. In addition, the dynamic oscillatory test showed that with increasing SPI concentration, the viscoelastic parameters (G’ and G”) of the starch network decreased. SPI could also prevent hardening of samples during 20 days of storage. In another study, Bartkiene et al. [68] prepared functional GCs formulated with lacto-fermented Vilciai and Vilniai lupine protein concentrates (LPCs). The *Lactobacillus sakei*-fermented LPCs from Vilniai variety showed the highest protein content (90.11%) and protein digestibility (89.94%), but the lowest trypsin inhibitor activity (19.40%). Furthermore, the incorporation of these proteins into the GC formulation up to 13.0%, not only affects the amino acid profile and genistein content of the samples, but also improves their texture and overall acceptability. In a different study, the possible application of 1, 3, and 5% *Spirulina* biomass as an innovative ingredient in the development of GCs was investigated by Paternina et al. [69]. They reported that the protein and mineral contents of *Spirulina*-enriched GCs increased by 36.7 and 414.3%, respectively, compared to the control sample. In addition, these functional GCs represented relatively high phenolic compounds (0.081 mg GAE/g), antioxidant activity (11.4%), and overall acceptability (80.0%). 

Overall, the addition of high-protein ingredients into GC formulations can be a promising way to obtain functional and nutraceutical confectionary products. However, there is still limited information available in this area and it needs to be given much more attention in the future work of food researchers.

### 3.3. High-Antioxidant GCs

Antioxidants are a group of natural or man-made substances that can prevent or slow damage to cells caused by free radicals, which are unstable molecules that the body produces as a reaction to environmental and other pressures. These substances can be found in many foods, including fruits and vegetables, and are also available as dietary supplements, such as vitamins C and E, selenium, and carotenoids. Additionally, it has been observed that numerous polyphenolic compounds present in herbal extracts possess notable antioxidant activities [70,71,72]. 

The incorporation of antioxidant substances in GC products has gained a lot of attention in recent years. For instance, Cedeño-Pinos et al. [62] conducted a study to assess the incorporation of rosemary extract into stevia-based GCs, aiming to enhance their antioxidant properties. The researchers found that adding 0.26 g of rosemary extract per kg of GC resulted in a significant increase in polyphenol content from 197 to 411 µg GAE/g and antioxidant capacity from 1.77 to 4.14 µmol Trolox/g. Importantly, this addition did not negatively impact the consumer acceptance of enriched GCs. Similarly, Charoen et al. [73] developed high-antioxidant GCs by utilizing *Psidium guajava* L. leaf extracts obtained at a drying temperature of 50 °C and a pH of 4.0. In another study by Archaina et al. [74], freeze-dried GCs using blackcurrant and yogurt were developed. The results showed that the antioxidant activity of both GC formulations was similar to kiwi and apple fruits (ranging from 4.2 to 4.5 mmol Trolox/kg), demonstrating the possible application of these components in the supplementation of GCs. In a further study, Ali et al. [24] evaluated the antioxidant properties of functional GCs incorporating fresh fruits, such as strawberries and red beetroots. GCs formulated with 75% strawberry and 25% beetroot exhibited the highest levels of bioactive compounds and antioxidant activity (52.55%) with acceptable sensory attributes. In addition, Altamash et al. [75] investigated the possible preparation of GCs using a blend of pineapple and beetroot juice. The GC prepared with a pineapple/beetroot ratio of 90:10 exhibited the best antioxidant and organoleptic qualities, indicating its favorable nutritional and sensory characteristics. These results are in line with the findings of A Abd EL Latif et al. [76], who developed GCs using natural plant extracts from lemons, red roselles, tamarind, peppermint, and dates. 

Furthermore, Gramza-Michalowska and Regula [77] utilized different concentrations of tea extracts (*Camelia sinensis*) in GCs as a source of polyphenolic compounds. The researchers observed that the polyphenol content and antioxidant activity of GCs increased with the addition of 1 and 1.5% tea extract, ranging from 245.9 to 1256.5 and 3.2 to 170.1 mg/100g, respectively. In another study, Suman et al. [78] showed a similar trend for GCs enriched with 1–6% ginger powder, 0.02–0.14% ginger oleoresin, and 5–30% ginger juice. In addition, Sarabandi and Mohammadi [79] evaluated the possible use of peppermint extracts in GCs and assessed their physical properties, total phenolic content (TPC), and antioxidant properties. The GC containing 10% peppermint extract exhibited the highest antioxidant capacity in various assays, making it a suitable antioxidant agent in the formulation of functional GCs with desirable texture, antioxidant, and sensory properties. In a further study, Ghendov-Mosanu et al. [80] substituted synthetic dyes with chokeberry extract in GCs. The researchers investigated the impact of different concentrations (i.e., 1.0, 1.5, and 2.0%) of chokeberry extract on the antioxidant and sensory properties of the candies. The results revealed that the extract improved the antioxidant capacity and sensory attributes of GCs at all studied concentrations, especially 1.5%. Therefore, the incorporation of natural antioxidants into the GC production can not only effectively improve the nutraceutical benefits of the final products, but also increase the buying intention of consumers.

### 3.4. GCs As a Medicine

In ancient times, it was mistakenly believed that the worse a medicine tastes, the better its therapeutic efficacy. Therefore, improving the taste of medicinal products was not considered by manufacturers. Today, the new perception of pharmaceutical technology is that the taste of an oral formulation should be sufficiently pleasant, especially when it is intended for pediatric use [81]. For this reason, several drug delivery systems have been developed in the past few decades. One of the most cost-effective and safe techniques is to incorporate drugs and bioactive compounds into GCs, which are gaining much more attention these days due to their favorable taste and structure, especially for children [82]. However, it is necessary to reformulate GCs according to the organoleptic and physicochemical properties of the active pharmaceutical ingredients, as well as the target consumers. In this regard, Karaiskou et al. [81] evaluated the possible incorporation of metoclopramide hydrochloride into the GCs containing pomegranate juice. According to the results, this technique met the pharmaceutical recovery requirements and can be used for routine consumption. Also, the stability of metoclopramide in the GCs was tested after 6 months of storage at 15 °C with satisfactory results. Such functional GCs can not only be considered for use by children, but also for adults who are facing various diseases such as mental illnesses. In another study, Hosseini et al. [83] successfully designed anti-fever and analgesic GCs based on gelatin and starch with nano-sized acetaminophen. Cumulative drug release of nanoparticles in GCs was approximately 74% after one day. These types of GCs can be the next generation dosage forms of acetaminophen due to their ease of consumption and greater compatibility among children than conventional oral tablets. However, more studies are needed to confirm present findings.

GCs can also be used for delivery of minerals and vitamins. In this respect, Handayani et al. [84] developed chitosan-based GCs loaded with ferrous gluconate (FeG) microparticles in order to increase the daily intake of iron. The authors demonstrated that the incorporation of FeG not only influenced the nutritional properties of GCs, but also affected their structural and textural characteristics during the storage period, in terms of shape, size, hardness and gumminess. In addition, Constantino and Garcia-Rojas [85] evaluated the functional and physicochemical properties of GCs enriched with beta-carotene (β-C) using amaranth carboxymethyl starch (CMS) and lactoferrin (LF). The results indicated that β-C was microencapsulated in CMS/LF complex coacervates up to 98% with favorable thermal and photolytic stabilities. Moreover, the encapsulation of β-C into GCs significantly protected about 66% of the β-C during processing, where temperatures reached over 100 °C. β-C-containing GCs also showed acceptable bioaccessibility (22%) and better textural properties (e.g., lower hardness). Therefore, the fortification of GCs with β-C microcapsules in CMS/LF complex coacervates can be considered as an effective way to overcome vitamin A deficiency worldwide. 

Development of GCs with probiotics and prebiotics is another emerging trend in today’s confectionery industry. For instance, Lele et al. [86] prepared functional GCs based on agar, gelatin, and apple pomace (as a pectin source) with the incorporation of probiotics (e.g., *Lactobacillus paracasei* LUHS244 and *Lactobacillus plantarum* LUHS135) and prebiotics (e.g., psyllium husk fibers). The antioxidant activities and textural properties of samples were strongly dependent on the strain of LAB and the use of psyllium husk fiber. Also, the enriched-GCs showed excellent antimicrobial properties against a wide range of pathogenic strains including *Escherichia coli*, *Streptococcus mutans*, *Streptococcus aureus*, *Enterococcus faecalis*, *Salmonella enterica*, *Proteus mirabilis*, and *Pseudomonas aeruginosa*. These results are in line with the findings of Bartkiene et al. [87], who developed probiotic GCs with the addition of bovine colostrum and various essential oils. The GCs held antimicrobial activities against different pathogenic bacteria such as *Escherichia coli*, *Streptococcus mutans*, *Enterococcus faecalis*, *Salmonella enterica*, *Proteus mirabilis*, *Pseudomonas aeruginosa*, and *Staphylococcus aureus*. They also reported that GCs consisting of 3% fermented bovine colostrum, 0.2% *Thymus vulgaris* L., *Citrus reticulate* L., and *Citrus paradise* L. essential oils represented the desired textural and sensorial properties, revealing the possible production of value-added GCs on an industrial scale. In a further study, Miranda et al. [88] evaluated the functional, physicochemical, and antioxidant properties of novel GCs reformulated with native fruits of the Atlantic Forest (i.e., palm Juçara (*Euterpe edulis* Martius) and passion fruit (*Passiflora edulis*)) and *Bacillus coagulans*. These GCs meet the probiotic survival requirements of laboratory testing recommended by Food and Agriculture Organization (FAO), as populations higher than 6.82 Log CFU/g reached the second viable intestinal phase, indicating the potential of the carrier matrix. These studies demonstrate the potential of probiotic and/or prebiotic GCs in the confectionary market due to their excellent biological and health-promoting benefits.

## 4. A Summary of Advantages and Disadvantages of Novel Functional GCs

Despite the techno-functional and health benefits of novel functional GCs, the incorporation of unconventional ingredients into formulations may cause some disadvantages, which are summarized in Table 1. However, there is still limited information available in most areas and more attention should be paid in the future.

## 5. Conclusions

The popularity of gummy candies (GCs) by children makes it worthwhile to develop more nutritious and health promoting products. Inclusion of healthier alternative ingredients such as natural flavors and colorants, natural and low-calorie sweeteners, plant and algal proteins, and dietary fibers into GCs is a promising approach to improve the low nutritional profile of this confectionary product. Moreover, GCs have potential utility as a delivery system for antioxidant compounds, plant extracts, and some types of medicines. The application of alternative components in GCs may contribute to their texture, color, flavor, sweetness level, and microbiological quality. These parameters should be considered when formulating functional GCs to fulfill consumer demands and expectations. Efforts should be made to develop GCs with superior nutritional and sensory properties.

## Figures and Tables

**Table 1:** Development of functional GCs with different strategies.

Novel GCs	Functional Ingredients	Advantages	Disadvantages
Gelatin-free	Pectin, guar gum, agar gum, carrageenan, carboxy methyl cellulose (CMC), alginate, corn starch, and cassava starch	□ Suitable for vegan and vegetarian diets □ Can be labeled as halal and kosher-certified products	▪ It is more difficult to control environmental factors such as pH compared to gelatin-based GCs ▪ Different hydrocolloids, such as pectin and agar gum, show a synergistic effect on the gel network ▪ Some alternative gelling agents require more sugar compared to gelatin to achieve the desired flavor
Sugar-free	Stevia, corn syrup, erythritol, xylitol, honey, isomaltulose, maltitol, isomaltitol, and natural sugar sources (e.g., grape, mulberry, and carob molasses)	□ A smaller amount may be needed to achieve the same level of sweetness compared to sugar □ Suitable for anti-diabetic and low-calorie diets	▪ Some alternative sweeteners may have a different mouthfeel or aftertaste than sugar ▪ Some alternative sweeteners may cause glucose intolerance
Artificial additives-free	- Natural fruit flavors (e.g., strawberry, raspberry, orange, lemon, pineapple, etc.) - Natural fruit colorants (e.g., blueberry, red beetroot, grape, etc.) - Anthocyanin, betanin, etc.	□ Natural additives can enhance the overall flavor and visual appeal of GCs □ Fruit flavors/colorants can also offer potential health benefits through their antioxidant properties	▪ Some natural colorants such as betanin are sensitive to heat and pH, and their color may deteriorate over time ▪ Natural food flavors and colorants may negatively affect the texture of GCs
High-fiber	- Fibers derived from fruits (e.g., grape, carambola starfruit, and papaya) - Non-starch polysaccharides (e.g., inulin and konjac glucomannan (KGM))	□ Higher dietary fiber content with potential prebiotic activity □ Better antioxidant and textural features that stay stable within processing	▪ Different dietary fibers may negatively affect the techno-functional properties of GCs
High-protein	Soy protein isolate (SPI), lupine protein concentrate (LPC), and *Spirulina* biomass	□ Better storage stability and overall acceptability □ Improved amino acid profile and genistein content	▪ There is limited information available in this area and it needs more attention in the future
High-antioxidant	Natural plant extracts (e.g., rosemary, *Psidium guajava* L. leaf, tea, peppermint, chokeberry, etc.), fresh fruits (e.g., strawberries and red beetroots), pineapple and beetroot juice, and ginger powder	□ Higher antioxidant activity and polyphenol content □ Natural antioxidants can not only effectively improve the nutraceutical benefits of the final products, but also increase the buying intention of consumers.	▪ There is limited information available in this area and it needs more attention in the future
Medicine	Metoclopramide hydrochloride, acetaminophen, ferrous gluconate (FeG), beta-carotene (β-C) probiotics (e.g., *Lactobacillus paracasei* LUHS244 and *Lactobacillus plantarum* LUHS135), prebiotics (e.g., psyllium husk fibers), and essential oils (e.g., *Thymus vulgaris* L., *Citrus reticulate* L., and *Citrus paradise* L.)	□ Pleasant taste as a drug carrier □ Greater compatibility among children than conventional oral tablets □ Better antimicrobial properties against a wide range of pathogenic strains	▪ There is still limited information available in this area and it needs more attention in the future
